# GENDER DIFFERENCES IN ANXIETY AMONG OLDER ADULTS: THE MEDIATION ROLE OF OUT-OF-HOME ACTIVITY

**DOI:** 10.1093/geroni/igad104.0942

**Published:** 2023-12-21

**Authors:** Lingming Chen, Elizabeth Procter-Gray, Linda Churchill, Annabella Aguirre, Jie Cheng, Qun Le, Scott Crouter, Wenjun Li

**Affiliations:** University of Massachusetts Lowell, Lowell, Massachusetts, United States; University of Massachusetts Lowell, Lowell, Massachusetts, United States; University of Massachusetts, Lowell, Lowell, Massachusetts, United States; University of Massachusetts Lowell, Lowell, Massachusetts, United States; University of Massachusetts Lowell, Lowell, Massachusetts, United States; University of Massachusetts Lowell, Lowell, Massachusetts, United States; The University of Tennessee Knoxville, Knoxville, Tennessee, United States; University of Massachusetts Lowell, Lowell, Massachusetts, United States

## Abstract

Research shows gender differences in anxiety. Given that men and women have different levels of out-of-home activity and out-of-home activity is associated with anxiety, this study examines if gender differences in anxiety are mediated by out-of-home activity among older adults. The prospective cohort study enrolled 303 women and men aged 65 to 95 years old in central Massachusetts (2018-2020). Anxiety was evaluated by the Beck Anxiety Inventory. The out-of-home activity was measured by the percentage of daily step counts outside the home, which was objectively assessed by accelerometer and Global Positioning System devices. Data were analyzed by using mediation analysis, controlling demographic (age, race/ethnicity), physical health (self-rated health, comorbidities), and mental health (depressive symptoms, perceived stress). Results showed that women reported significantly greater scores in anxiety and did less out-of-home activity than men. Furthermore, out-of-home activity was significantly associated with anxiety. With the bootstrapping procedure, a significant indirect effect of gender on anxiety through out-of-home activity was shown (indirect effect: 0.025; 95% CI: 0.002-0.078), which explained 12% of the total effect (total effect: 0.214; 95% CI: 0.032-0.396). In conclusion, out-of-home activity partially mediates gender differences in anxiety. This study indicates that increasing out-of-home activities may help reduce anxiety and gender differences in anxiety among older adults.

